# Functional genomics for breast cancer drug target discovery

**DOI:** 10.1038/s10038-021-00962-6

**Published:** 2021-07-20

**Authors:** Tetsuro Yoshimaru, Yusuke Nakamura, Toyomasa Katagiri

**Affiliations:** 1 Division of Genome Medicine, Institute of Advanced Medical Sciences, Tokushima University, Tokushima, Japan; 2 Cancer Precision Medicine Center, Japanese Foundation for Cancer Research, Koto-ku, Tokyo Japan

**Keywords:** Drug discovery, Cancer

## Abstract

Breast cancer is a heterogeneous disease that develops through a multistep process via the accumulation of genetic/epigenetic alterations in various cancer-related genes. Current treatment options for breast cancer patients include surgery, radiotherapy, and chemotherapy including conventional cytotoxic and molecular-targeted anticancer drugs for each intrinsic subtype, such as endocrine therapy and antihuman epidermal growth factor receptor 2 (HER2) therapy. However, these therapies often fail to prevent recurrence and metastasis due to resistance. Overall, understanding the molecular mechanisms of breast carcinogenesis and progression will help to establish therapeutic modalities to improve treatment. The recent development of comprehensive omics technologies has led to the discovery of driver genes, including oncogenes and tumor-suppressor genes, contributing to the development of molecular-targeted anticancer drugs. Here, we review the development of anticancer drugs targeting cancer-specific functional therapeutic targets, namely, MELK (maternal embryonic leucine zipper kinase), TOPK (T-lymphokine-activated killer cell-originated protein kinase), and BIG3 (brefeldin A-inhibited guanine nucleotide-exchange protein 3), as identified through comprehensive breast cancer transcriptomics.

## Introduction

Breast cancer is the most common cancer among women worldwide [1, 2]. It is a heterogeneous disease [3] categorized into three main intrinsic subtypes based on expression of hormone receptors (HRs: estrogen receptor (ER) and progesterone receptor (PgR)) and human epidermal growth factor receptor 2 (HER2): HR-positive/HER2-negative (luminal-type: >70%), HER2-positive (15–20%) and HR- and HER2-negative (triple-negative BC; TNBC: 15%) [4, 5]. Current endocrine-based treatments targeting estrogen (E2)-ERα signaling (selective estrogen receptor modulators, selective estrogen receptor downregulation, aromatase inhibitors) [6, 7] as well as some molecular target drugs, such as mTOR inhibitors [8, 9], PI3K inhibitors [10], and CDK4/6 inhibitors [11–14], have been clinically applied for treating luminal-type breast cancer. For those with HER2-positive breast cancer, therapies involving drugs that target HER2, including trastuzumab [15, 16], pertuzumab [17], trastuzumab-emtansine [18, 19], and tyrosine kinase inhibitors of HER2, such as neratinib [20], pyrotinib [21], tucatinib [22, 23], and trastuzumab deruxtecan, which were recently approved for HER2-positive breast cancer [24], are currently being applied and can lead to significant improvement in survival. Nevertheless, the efficiency of these treatments, including endocrine and anti-HER2 therapies, is limited by common intrinsic and acquired resistance and the occurrence of adverse effect. In particular, TNBC patients do not benefit clinically from endocrine or anti-HER2 therapies by the lack of receptor expression (ER, PgR, and HER2) for drugs, and chemotherapy is the only standard drug treatment established to date. Recent detailed analysis based on gene expression profiling has revealed that TNBCs can be classified into at least six subtypes [25], including two basal-like (BL1 and BL2), immunomodulatory (IM), mesenchymal (M), mesenchymal-stem like, and luminal androgen receptor (LAR) subtypes. BL1 and BL2 tumors which carry BRCA1/2 mutations are genomically unstable due to defective of homologous recombination repair [25]. Of note, olaparib, an oral PARP inhibitor, has been approved for TNBC, HER2-negative metastatic breast tumors with germline BRCA-mutated (gBRCAm) [26, 27]. In addition, it is applicable for gBRCAm patients with hormone receptor-positive breast cancer who had received endocrine therapy [26, 27]. Thus, because TNBC and HER2-negative breast cancer patients with gBRCAm are rare, developing novel effective therapeutics for all subtypes of TNBC and HER2-negative breast cancers has become an urgent issue. Moreover, new approaches for therapeutic or diagnostic interventions in preclinical and clinical studies are needed, and molecules involved in the initiation, progression, and metastasis of TNBC may become targets for the treatment of this disease.

In recent years, comprehensive cancer omics data have become increasingly important in clinical practice for elucidating detailed molecular mechanisms and for the development of new cancer diagnostic and therapeutic modalities. Of note, analyses of cancer genome and transcripts by next-generation sequencing using large-scale cancer clinical cohorts have allowed to establish several databases such as The Cancer Genome Atlas (TCGA) led by the U.S. National Institutes of Health (NIH), the International Cancer Genome Consortium (ICGC), and Catalog of Somatic Mutations in Cancer (COSMIC), have been established. Based on sequencing data for thousands of cases, these cohorts were constructed using clinical information for each case, including single-gene mutations, genomic structural abnormalities (e.g., chromosome copy number abnormalities), genomic DNA methylation data, and mRNA- and protein-level expression data. These databases are essential for achieving a foothold for new research and obtaining data to support research, as opposed to the conventional method of testing hypotheses based on individual researchers’ knowledge and experience.

In general, transcriptomics through next-generation sequencing- and microarray-based technologies contributes to a detailed characterization of the nature of breast carcinogenesis and progression to improve clinical strategies for treatment through novel anticancer drug discovery, providing a basis for precision medicine. To reach such goals, we analyzed gene expression profiles for breast cancer and healthy tissues obtained by transcriptomics to identify cancer-specific functional molecules for the development of drugs [28–30]. Nonetheless, obtaining accurate and comprehensive transcriptome analysis data has remained challenging because of the high tissue heterogeneity of breast cancer. In particular, adipocytes have mostly been used as normal breast tissue controls. Hence, to obtain precise transcriptomics data for breast carcinogenesis and progression, we performed microdissection under a microscope to selectively collect breast cancer cells and normal ductal epithelial cells and then extracted total RNA from each and analyzed their transcriptomics data.

Furthermore, we adopted strategies to select “therapeutic target genes” for breast cancer therapy-based analysis of gene expression information obtained by the cDNA microarray method, with which we were able to screen genes showing upregulation in breast cancer cells compared to normal breast ductal cells dissected from clinical breast cancer tissue sections [28, 30]. To avoid severe side effect from therapeutic drugs, we screened genes with low expression in vital organs such as the heart, lungs, liver, and kidney based on gene expression profiling analysis of normal human organs. Subsequently, we selected genes for which RNA interference-mediated knockdown in breast cancer cell lines was effective at suppressing cell proliferation (sphere or organoid formation), migration, and invasion. In this step, a current advanced strategy using shRNA- or CRISPR/Cas9-based systems combined with next-generation sequencing analyses [31–33] helped to identify therapeutic target genes and signaling axes regulated by the oncogene addiction phenotype in breast cancer cells. By applying a combination of these approaches, we ultimately identified and characterized more than a dozen “cancer-specific functional genes” of biological and medical importance [34–45] (Table 1). Here, we focus on three cancer-specific therapeutic target molecules: two kinases, namely, maternal embryonic leucine zipper kinase (MELK) and T-lymphokine-activated killer cell-originated protein kinase (TOPK); and one scaffold anchoring protein, namely, brefeldin A-inhibited guanine nucleotide-exchange protein 3 (BIG3). We also review the development of novel anticancer drugs targeting these cancer-specific molecules for breast cancer therapy currently in preclinical or clinical use.

**Table 1:** Promising cancer-specific functional targets for breast cancer therapy

Genes	Gene ID (NCBI)	Functions	Expression data in breast cancer cases (%)^a^	Expression data in normal human organs	Ref.
*MELK*	9833	Serine/threonine kinase	26/34 (76.5%)	Testis	[33]
*TOPK*	55872	Serine/threonine kinase	31/40 (77.5%)	Testis, thymus, spleen,	[34]
*BIG3*	57221	Anchoring scaffold protein	26/41 (63.4%)	Pancreas, brain	[35]
*C12orf32*	83695	DNA damage response	24/78 (30.8%)	Testis, prostate	[36]
*DTL*	51514	Mitotic cell regulator	54/79 (68.4%)	Testis, placenta thymus	[37]
*GALNT6*	11226	UDP-N-acetyl-alpha-D-galactosamine:polypeptide N-acetylgalactosaminyltransferase	30/81 (37.0%)	Placenta, pancreas, stomach,	[38]
*GPATCH2*	55105	Enhancer of ATPase activity	17/42 (40.5%)	Testis,	[39]
*GPSM2*	29899	Modulator of G proteins activation	16/22 (72.7%)	Brain, lung	[40]
*KIF2C*	10112	Mitotic cell regulator	47/63 (74.6%)	Testis, bone marrow, thymus	[41]
*PRC1*	9055	Mitosis regulator (cytokinesis)	37/59 (62.7%)	Testis, bone marrow, thymus	[42]
*RQCD1*	9125	Signal transduction regulator	11/14 (78.6%)	Testis	[43]
*UBE2T*	09089	E2 Ubiquitin ligase	41/50 (80.2%)	Testis, skeletal muscle	[44]

^a^Breast cancer patients who show the upregulation (>5-fold) of each gene in breast cancer cells compared with normal breast epithelial cells [27]

### MELK

Most of the small molecule-targeted anticancer drugs being applied are kinase inhibitors. MELK is a member of the snf1/AMPK serine-threonine kinase family, the members of which are involved in regulating various kinds of cellular events, including mammalian embryonic development [46–49]. MELK is significantly upregulated in many human cancers, including breast cancer [28, 30, 50–52], but it exhibits low or undetectable expression in normal organs, except the testis. Notably, through gene expression profiling analyses, we and others have reported that MELK is markedly upregulated in TNBC and basal-like breast cancer [30, 53]. Indeed, MELK activation plays a critical role in various cellular events, including hematopoiesis, stem cell renewal, cell cycle progression, and apoptosis regulation, through interaction with the zinc finger-like protein ZPR9, splicing factor NIPP1 and proapoptotic factor Bcl-GL [34, 54–56]. Among them, Bcl-GL was identified as a critical substrate in screens involving in vitro protein pull-down assays using wild-type MELK and recombinant kinase-dead MELK. The findings revealed that MELK physically interacts and phosphorylates Bcl-GL and that MELK-mediated phosphorylation of Bcl-GL suppresses its proapoptotic activity. Overexpression of wild-type MELK suppresses Bcl-GL-induced apoptosis, whereas a kinase-dead MELK mutant does not [34]. Moreover, by inducing the proapoptotic function of Bcl-GL, siRNA-mediated depletion of MELK expression significantly inhibits the growth of human breast cancer cells [34]. Chung et al. reported that MELK is indispensable for promoting and maintaining cancer stem cells (CSCs) via upregulation of the stem cell marker Oct3/4 [57]. MELK was also found to be upregulated in tumor-initiating cells isolated from a genetically engineered mouse model of breast cancer, indicating that MELK function might be required for breast carcinogenesis via the development and maintenance of CSCs. Moreover, MELK appears to regulate the stability of the oncogene product DEPDC1 through phosphorylation. As DEPDC1 is involved in cytoskeletal regulation and brain metastasis, MELK might be involved in brain metastasis of breast cancer via DEPDC1 regulation [58].

**Fig. 1:**
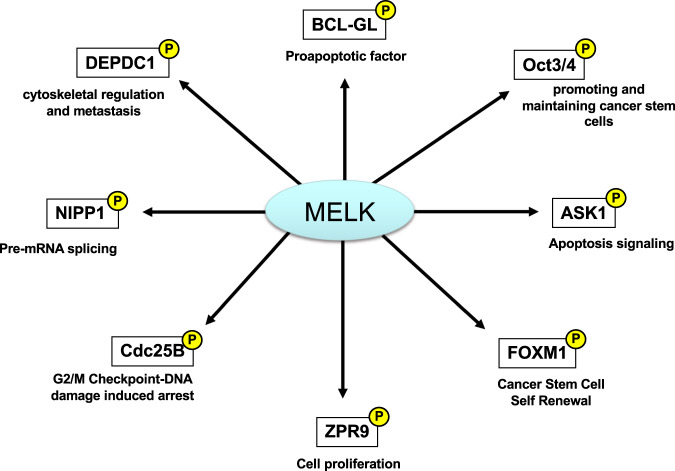
The MELK kinase is involved in several biological functions

Additionally, MELK is reportedly activated by various kinds of cellular stresses, including transforming growth factor-β (TGF-β), apoptosis signal-regulating kinase 1 (ASK1), p53 signaling pathways, and the transcription factor forkhead box protein M1 (FOXM1), resulting in cell cycle and apoptosis regulation and proliferation enhancement [50, 59–62]. Collectively, these findings suggest that MELK expression is upregulated in breast cancer, especially in TNBC, and is a promising diagnostic and therapeutic modality. Overall, MELK is reportedly involved in several biological functions (Fig. 1).

Recently, MELK knockout by the CRISPR/Cas9 system was reported to have no effect on cancer cells [63]. Nevertheless, six studies reported that exogenous MELK expression was able to rescue the effect of growth suppression, tumorsphere suppression, or cell death by RNAi-mediated MELK knockdown in cancer cells [53, 64–68]. Of note, two studies showed that a MELK kinase-dead mutant was not able to rescue the effect of MELK knockdown in cells [53, 65]. This evidence strongly suggests that MELK, especially its kinase activity, is necessary for the proliferation of cancer cells. In addition, off-target effect arise from both CRISPR/Cas9 system-mediated knockout and RNAi-mediated knockdown. However, RNAi-mediated knockdown and subsequent rescue experiments mitigated the off-target effect of RNAi. In RNAi-mediated knockdown studies, multiple siRNAs (shRNAs) targeting MELK mRNA were designed to avoid off-target effect, with their on-target effect on MELK expression confirmed [34, 53, 66, 68]. Accumulating evidence shows that MELK is upregulated under cell cycle dependency in various cancers, including breast cancer, even though CRISPR/Cas9-mediated MELK knockout has no effect on cancer cell proliferation. In this regard, McDonald IM and Graves LM mentioned in their review the possibility that MELK is functionally redundant for a specific cell cycle pathway, such that MELK is not indispensable for the cell cycle in normal cells but is necessary for specific conditions in cancer cells [69]. On the other hand, CRISPR/Cas9-mediated MELK knockout may allow for compensatory signaling networks as MELK functional redundancy, whereas RNAi-mediated MELK knockdown does not allow for the possible redundant pathway [69]. Collectively, despite strong controversy, these findings suggest that MELK is necessary for the growth of cancer cells, especially breast cancer cells.

### Development of MELK inhibitors

Several MELK inhibitors have been developed (Table 2). For example, Chung et al. reported that high-throughput screening of a library consisting of more than 100,000 small compounds followed by structure-activity relationship (SAR) studies resulted in the development of the highly potent MELK inhibitor OTS167 (also known as OTSSP167). OTS167, with an IC50 of 0.41 nM, comprises a 1,5-naphthyridine core with a methyl ketone at the 3-position, trans-4-(dimethylamino) methyl) cyclohexyl amino at the 4-position, and 3,5-dichloro-4- hydroxyphenyl at the 6-position [58] (Table 2). Recent studies have shown that treatment with OTS167 drastically suppresses the growth of various MELK-overexpressing cancer cell lines, including breast, lung, prostate, AML, gastric, kidney, and ovarian cancers [51–53, 58, 70]. By suppressing phosphorylation of its substrates proteasome subunit alpha type 1 and drebrin-like, OTS167 treatment also inhibits tumor cell invasion and mammosphere formation by breast cancer cells [58]. More importantly, intravenous administration of OTS167 at 20 mg/kg once every 2 days caused significant tumor growth inhibitory (TGI) effect in breast cancer (MDA-MB-231) xenograft mice, without body weight loss or other toxicities at effective doses (Table 2). In addition, the high bioavailability of OTS167 allows for oral administration of OTS167 at 10 mg/kg once a day, with significant TGI effect of 72% and 124% in MDA-MB-231 TNBC and A549 lung cancer xenograft mice, respectively [58]. OTS167 is currently being assessed in two phase I clinical trials of solid tumors (Clinical Trial No. NCT01910545) and hematologic malignancies (Clinical Trial No. NCT02795520). Moreover, OTS167 was recently reported to inhibit BUB1 and Haspin kinases as well as MELK, reducing phosphorylation at histones H2A and H3 and causing mislocalization of Aurora B, which resulted in abolishment of the mitotic checkpoint and abortion of cytokinesis in cancer cells [71].

**Table 2:** Antitumor effect of MELKi, TOPKi. and ERAP on breast cancers

Inhibitors	Structure	Enzyme IC50	Growth suppressive effect in BC cell lines in vitro and in vivo	Ref.
OTS167		MELK: 0.41 nM	DU4475 (TNBC); 2.3 nM MDA-MB-231 (TNBC); 22.0 nM SUM-159 (TNBC); 67.3 nM MDA-MB-468 (TNBC);14 nM (3-day) BT-549 (TNBC); 21 nM (3-day) HCC70 (TNBC); 34 nM (3-day) T47D (luminal); 4.3 nM (106 nM;3-day) MCF-7 (luminal) 35 nM (3-day) **MDA-MB-231 (25** **mg/kg) in vivo** **MDA-MB-468 (5** **mg/kg) in vivo** **MCF-7 (5** **mg/kg) in vivo** **T47D(5** **mg/kg) in vivo**	[52, 56]
MELK8a		MELK:11.9 nM	MDA-MB-468 (TNBC);5.41 nM (3-day) BT-549 (TNBC); 8.05 nM (3-day) HCC70 (TNBC); 5.99 nM (3-day) T47D (luminal); >10 nM (3-day) MCF-7 (luminal) 6.06 nM (3-day) ZR-75-1(luminal); >10 nM (3-day)	[52, 63]
Compound 17		MELK:0.39 nM	MCF-10A no effect at 10 µM HCC70 (TNBC); >1.0 μM BT-549(TNBC);>1.0 μM	[64]
OTS514		TOPK:2.6 nM	DU4475 (TNBC); 14 nM MDA-MB-231 (TNBC); 14 nM T47D (luminal); 14 nM	[67]
OTS964		TOPK:28 nM	DU4475 (TNBC); 53 nM MDA-MB-231 (TNBC); 73 nM T47D (luminal); 72 nM	[67]
stERAP		BIG3-PHB2: Kd 3.52 μM	MCF-7 (luminal);1.02 μM KPL3C (luminal); **10 mg/kg (weekly)** in vivo	[89]

The bold letters indicate the in vivo tumor efficacy

Other potent small molecule compound MELK inhibitors, namely, MELK 8a and compound 17, were identified through a combination of high-throughput and virtual screening strategies and kinase inhibitor library screening, respectively. MELK 8a treatment selectively suppresses MELK-positive MDA-MB-468 breast cancer cell growth [72]. Compound 17 has also been identified as a potent MELK catalytic domain inhibitor through kinase library screening and structure-guided design of a series of ATP-competitive indolinone derivatives. Treatment with compound 17 inhibits the growth of MELK-positive TNBC cell lines HCC70, BT-549, and SUM159 via regulation of MCl-1, an antiapoptotic protein of the BCL-2 family [73].

### TOPK

TOPK is also a serine/threonine-protein kinase that is highly upregulated in multiple cancer types, such as leukemia and breast, kidney, and ovarian cancers [30, 35, 52, 74, 75]. Conversely, TOPK expression is hardly detectable in normal organs, except for the testis, with a low level in the thymus [35]. Immunohistochemical analysis has confirmed intense staining in cells of breast cancer tissues; in normal organs, staining was observed in the testis but not in normal mammary duct cells or normal vital organs (heart, liver, kidney, or lung) [35]. Importantly, Kaplan–Meier survival analyses have shown that higher TOPK expression correlates significantly with a poor prognosis for breast and lung cancer patients, suggesting that TOPK inhibitors can improve the clinical outcome of these patients with high TOPK expression [76]. In contrast, knockdown of TOPK expression significantly inhibits the growth of breast, AML, kidney, and ovarian cancer cells, leading to abnormal cytokinesis, apoptosis induction and marked cell proliferation inhibition [35, 52, 75, 76].

**Fig. 2:**
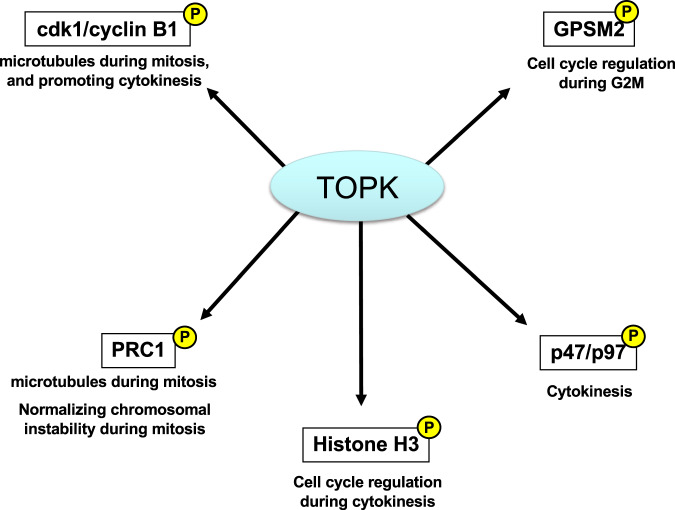
The TOPK kinase is involved in several biological functions

TOPK is activated in a cell cycle-dependent manner, especially in early mitosis; this is followed in mid-mitosis by phosphorylation of its tenth serine residue using histone H3 as a substrate, which indicates its proliferation-promoting effect [35]. Furthermore, TOPK is highly activated by autophosphorylation during late mitosis, and suppression of its function abrogates cytokinesis in cancer cells, leading to the expansion of intercellular bridges between two dividing cells followed by apoptosis [35]. We identified the p47/p97 complex as another substrate of TOPK by a pull-down assay and found that p97, an ATPase, is directly phosphorylated by recombinant TOPK [77]. In addition, p97 knockdown led to cytokinesis failure in breast cancer cells, similar to the effect of TOPK knockdown. The p97 protein regulates spindle disassembly at the end of mitosis and acts as a critical coordinator of cellular morphology from M to the next G1 phase. According to Abe et al. TOPK phosphorylates and then binds to cdk1/cyclin B1 and protein regulator of cytokinesis 1 (PRC1) on microtubules during mitosis to promote cytokinesis [78]. Overall, these findings suggest that TOPK functions as an essential mitotic kinase through phosphorylation depending on the regulation of substrates involved in cytokinesis, including the p47/p97 complex, histone H3, cdk1/cyclin B1, and PRC1. Thus, TOPK kinase is involved in several biological functions (Fig. 2).

### Development of TOPK inhibitors

Matsuo et al. reported the development of the potent TOPK inhibitors OTS 514, {(R)-9-(4-(1-aminopropan-2-yl) phenyl)-8-hydroxy-6-methylthieno[2,3-c]quinolin-4(5H)-one) and OTS-514 analog compounds OTS964, {(R)-9-(4-(1-(dimethylamino) propan-2-yl) phenyl)-8-hydroxy-6-methylthieno[2,3-c]quinolin-4(5H)-one} via high-throughput screening of a small molecule compound library, extensive SAR studies and MELK inhibitors [76] (Table 2). These compounds have strong potential to inhibit TOPK kinase activity, with IC50 values of 2.6 and 28 nM for OTS514 and OTS964, respectively. For example, treatment with OTS514 markedly inhibits the growth of many TOPK-positive cancer cell lines, with low IC50 values ranging from 1.5 to 48.7 nM. Similar to the effect of TOPK knockdown in breast cancer cells, OTS514 treatment leads to cytokinesis failure, with intercellular bridge elongation between dividing daughter cells through inhibition of TOPK activity [76]. Importantly, although intravenous administration of OTS514 results in significant antitumor effect in lung cancer xenograft mice, hematopoietic toxicity also occurs. Matsuo et al. attempted to improve the safety and efficacy of TOPK suppression by developing liposomal OTS964, a dimethylated derivative of OTS514 with enhanced bioavailability. After five shots of liposomal OTS964 treatment twice per week, treatment with OTS964 led to complete regression in five of six tumors, without any of the hematopoietic toxicity caused by OTS514. Moreover, oral administration of OTS964 without liposomes led to complete regression of tumors in all mice treated [67]. Although the treatment also caused some hematopoietic toxicity, all mice recovered spontaneously after the termination of treatment. Other studies have reported antitumor effect of OTS514 or OTS964 in mouse xenograft models of AML, ovarian cancer, multiple myeloma, kidney and small cell lung cancers [35, 52, 74, 75, 79] (Table 2).

Another potent TOPK inhibitor, ADA-07 (5-((1s, 3s)-adamantan-1-yl)-3-(hydroxyimino) indolin-2-one), was developed for treatment of solar ultraviolet (SUV)-induced skin carcinogenesis. ADA-07 directly binds within the ATP-binding pocket of TOPK and suppresses its kinase activity as well as activation of the TOPK downstream molecule ERK1/2. P38, JNKs and then MAPK signaling pathway [80, 81]. Another TOPK inhibitor, HI-TOPK-032, displays antitumor effect in colon and SUV-induced skin cancers in vitro and in vivo and nasopharyngeal xenograft mouse tumors in vivo [82, 83].

### BIG3

**Fig. 3:**
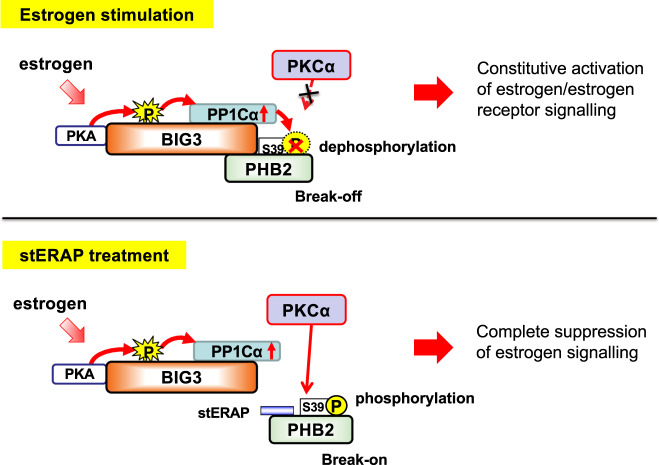
Pathophysiological role of the BIG3-PHB2 complex in estrogen-dependent breast cancer cells. Estrogen (E2) stimulation induces PKA-dependent BIG3 phosphorylation, which cancels its negative regulation of PP1Cα activity, resulting in the avoidance of PHB2 suppressive activity (Upper panel). stERAP competitively binds to endogenous PHB2, thereby preventing its interactions with BIG3. Free PHB2 directly binds to both nuclear and plasma membrane-associated ERα, resulting in repression of E2-induced genomic and non-genomic ERα activation (Lower panel)

BIG3 (also known as ARFGEF3) is a member of the BIG/Sec7p subfamily of ADP ribosylation factor-GTP exchange factors. BIG1 and -2 are also members of the BIG/Sec7p subfamily and contain highly conserved ec7 domains that catalyze the replacement of ARF-bound GDP by GTP to initiate membrane vesicle formation [84–87]. Although BIG3 is considered to belong to the sec7/Arfs protein family, it contains a single highly conserved Sec7 domain and shares only 25% identity in amino acid sequence with BIG1 and BIG2 [84–87], indicating that BIG3 is a protein with unknown physiological function. Nevertheless, BIG3 is exclusively overexpressed in the majority of breast cancers and exhibits extremely low expression in most normal organs, except for the pancreas and brain [36]. Knocking down BIG3 drastically reduces the growth of multiple breast cancer cell lines, and immunohistochemical staining of ERα-positive breast cancer clinical specimens has revealed BIG3 positivity in ~90% of cases [88, 89]. Importantly, patients with strong BIG3-positive staining have shorter disease-free survival than those with negative/weak expression [88]. Furthermore, statistical analysis of public databases has confirmed that BIG3 correlates with prognosis in all subtypes of luminal-type breast cancer, suggesting its contribution to the progression and malignancy of the disease. We also demonstrated that BIG3 functions as an A-kinase anchoring protein that forms a complex with protein kinase A (PKA) and the alpha-isoform of the catalytic subunit of protein phosphatase 1 (PP1Cα) through their binding motifs, which were identified by in silico analysis [88]. BIG3 also binds to prohibitin 2 (PHB2) [36, 88–90], which is known to function as a repressor of many cancer-related pathways, including estrogen signaling [91, 92]. We found that PHB2 Ser39 phosphorylation is essential for its suppression of E2-induced genomic and nongenomic ERα signaling activation [88, 89]. BIG3 phosphatase activity maintains PHB2 in a dephosphorylated inactive state, leading to an apparent “loss-of-function” PHB2 protein. These findings indicate that the existence of a loss-of-function mechanism of the innate tumor suppressor PHB2 is essential for malignant transformation in breast cancer (Fig. 3).

### Development of peptide inhibitors targeting the BIG3-PHB2 interaction

The existence of the PHB2 loss-of-function mechanism by the cancer-specific molecule BIG3 will contribute to novel drug discovery based on reactivation of the innate suppressive function of PHB2. Therefore, we employed the protein–protein interaction site prediction server (PSIVER) method [93, 94], a computational prediction system to predict residues that bind to other proteins using only sequence features, to identify protein binding sites of BIG3. The region encompassing amino acid positions 101–250 on the α-helix structure of BIG3 was identified as a cluster of candidate binding residues, particularly the side chains of Gln165, Asp169, and Gln173, which have the highest scores and are oriented in the same direction [89, 93, 94]. Indeed, BIG3 mutants in which all of these target residues are substituted with alanine showed almost no interaction with PHB2, indicating the importance of the region in the vicinity of these three amino acids for BIG3 dimerization with PHB2. Accordingly, we focused on 13 residues (165–177: QMLSDLTLQLRQR) of the alpha-helix structure and synthesized the dominant-negative peptide “ERAP (ERα activity-regulator synthetic peptide)”. This peptide consists of these 13 amino acids and plasma membrane-permeable polyarginine residues (11R) at the NH_2_ terminus [89]. Treatment of breast cancer cell lines with ERAP led to rapid dissociation of PHB2 captured by BIG3 and thereby rapid, E2-dependent PHB2 phosphorylation at Ser39, allowing reactivation of the innate suppressive capability of PHB2. Phosphorylated PHB2 translocates to nuclear ERα and plasma membrane-type ERα in an E2-dependent manner, resulting in multiple suppression of E2-dependent transcriptional activity and membrane-type ERα-mediated nongenomic signaling (various phosphorylation cascades) in ERα-positive breast cancer [89, 95, 96]. Similar findings have also been reported for orthotopic breast cancer xenografts in nude mice [88, 89, 97]. The most notable aspects of this therapeutic strategy are that (1) PHB2 reactivated by ERAP suppresses various E2-ERα signaling networks, which are responsible for resistance to endocrine therapy, and (2) ERAP has antitumor effect on endocrine-resistant breast cancer xenografts [89]. Notably, the mechanism of action of ERAP does not affect E2 production, resulting in significant avoidance of the menopause-like adverse effect associated with current endocrine therapy. Collectively, it is expected that a therapeutic strategy targeting the interaction between BIG3 and PHB2 will be effective for breast cancer, with few serious adverse effect.

Considering its clinical use, it is essential to improve the proteolytic stability of ERAP to better maintain its inhibitory activity. Focusing on the fact that the relevant amino acid sequences of ERAP form an alpha-helix structure, we developed chemically modified ERAP (hereafter referred to as stapled ERAP: stERAP) via cross-linking of specific amino acids, with amino acids essential for BIG3-PHB2 interactions (Gln165, Asp169, and Gln173) spatially arranged at appropriate positions [97]. stERAP led to enhanced stabilization of the alpha-helical structure, potential protease resistance, and enhanced cell permeability, without the membrane-permeable polyarginine sequence of conventional ERAP [97]. As a result of these properties, stERAP achieved E2-dependent responsiveness with long-term stability, exhibiting antitumor effect after weekly intravenous administration (10 mg/kg body weight) in clinical application (Table 2) [97]. We further demonstrated that stERAP potentiates antitumor activity in clinically problematic endocrine-resistant breast cancer. Notably, we showed that stERAP leads to synergistic inhibitory effect when in combination with existing anti-hormonal agents (tamoxifen and fulvestrant) or molecular-targeted drugs (everolimus) [36]. These achievements highlight the excellent therapeutic benefits achieved through reactivation of the tumor suppressor PHB2 and suggest the possibility of new therapeutics that will replace conventional endocrine therapeutics targeting E2 signaling.

## Conclusion

Most existing anticancer drugs have very low selectivity for cancer cells and are expected to exert anticancer effect through cytotoxicity. Due to their mechanism of action, these drugs have strong side effect on normal cells, especially bone marrow cells, which have a short cell cycle. The identification of cancer-specific molecules by comprehensive omics, particularly transcriptomics, will not only help to elucidate the mechanisms of breast carcinogenesis and progression but will also lead to the development of molecularly targeted therapeutics that provide greater efficacy with fewer side effect in many patients. Of note, modulation of protein–protein interaction inhibitors (PPIs) using peptides, compounds and antibodies is a major challenge for next-generation drug discovery owing to problems such as production costs and intracellular delivery systems. Recently, the application of peptides as new PPI factors to replace proteins has attracted attention. In this review, we introduce three promising molecular targets, MELK, TOPK, and BIG3, that are specifically overexpressed in many cancers, including breast cancer. Overall, cancer-specific functional molecules and interactions can be effectively and selectively inhibited by small compounds or dominant-negative peptides. In particular, the discovery that stERAP enables reactivation of the innate tumor-suppressive activity of PHB2 based on this molecular mechanism may lead to therapeutics that greatly contribute to the treatment of refractory breast cancer without impairing the quality of life of patients.
